# Bovine Breeds Identification by Trichological Analysis

**DOI:** 10.3390/ani9100761

**Published:** 2019-10-02

**Authors:** Gisele Aparecida Felix, Maria Clorinda Soares Fioravanti, Martino Cassandro, Nicola Tormen, Juliana Quadros, Raquel Soares Juliano, Andrea Alves do Egito, Maria Ivete de Moura, Ubiratan Piovezan

**Affiliations:** 1 School of Veterinary Medicine, University Centre of Grande Dourados Region, Dourados 79824-900, Brazil; 2 School of Veterinary Medicine and Zootechny, Federal University of Goiás, Goiânia 74690-900, Brazil; mariaclorinda@gmail.com (M.C.S.F.); medivetemoura@yahoo.com.br (M.I.d.M.); 3 Department of Agronomy, Food, Natural resources, Animals and Environment, University of Padova, 35020 Legnaro, Italy; martino.cassandro@unipd.it; 4 Department of Biology, University of Padova, 35131 Padova, Italy; nicola.tormen@gmail.com; 5 Department of Biology, Federal University of Paraná, Curitiba 83260-000, Brazil; quadros.juliana@hotmail.com; 6 EMBRAPA Pantanal, Corumbá 79320-900, Brazil; raquel.juliano@embrapa.br; 7 EMBRAPA Gado de Corte, Campo Grande 79106-550, Brazil; andrea.egito@embrapa.br; 8 EMBRAPA Tabuleiros Costeiro, Aracaju 49025-040, Brazil; ubiratan.piovezan@embrapa.br

**Keywords:** animal genetic resources, breed marker, genetic characterization, guard hair, non-invasive

## Abstract

**Simple Summary:**

This study examined the hair of Brazilian local cattle breeds in order to verify the possibility of use this technique as a racial marker. The trichological analysis is a non-invasive method that allows the determination of the species, or even the breed, of mammals by means of the combination of the main microstructures of the hairs. This kind of analysis has been used to identify species in ecological, paleontological, archaeological, contaminating materials of human food and forensic issues, but has been little explored in agriculture.

**Abstract:**

This study aimed to identify bovine breeds through trichological morphology and morphometry and to validate this technique by comparing it with genetic characterization. Animals from Caracu, Curraleiro Pé-Duro, Nelore, and Bovino Pantaneiro breeds were studied. Morphological and morphometric analyses of the guard hairs were performed. The cuticular pattern was observed on the shaft and the medulla pattern on the shield of the samples. The cattle genetic characterization was accomplished using microsatellite markers. Statistical analyses were performed using R version 3.2.4 software. Pearson’s correlation test showed a high positive and significant correlation between the matrices generated by trichological and genetic analyses (*r* = 0,996 and *p* < 0.001). Trichological analysis is a useful method for cattle breed identification. Its potential for identifying other species of interest for animal production should be studied since it is a simple, low-cost, and non-invasive method.

## 1. Introduction

The demand for animal products has increased, generating competition for resources and expanding the productivity and efficiency of livestock systems. Animal genetic resources (AGR) are essential for global food security and for the conservation of distinct livelihoods [1]. In Brazil, the local cattle are considered less productive under certain conditions, although they have unique characteristics of both rusticity and survival that may be necessary to ensure adaptive potential in the future [2].

Products with geographical signs in Europe are listed as examples of policy to expand the competitiveness of products from local breeds, emphasizing their differentiation and qualification [2]. The characterization of local breeds is a mandatory step for AGR conservation. This provides information to elucidate important issues related to rusticity, prolificacy, resistance, and adaptation to adverse conditions, which justifies the conservation programs [3]. Molecular markers have revolutionized our ability to characterize genetic variation [4]. However, the routinely use of this method can be limited by cost, laboratory efforts, and skilled labor. 

The hypothesis of the study is that trichological analysis is a viable alternative for the characterization of breeds in the productive context, being as efficient as a use of molecular markers for species differentiation and presents low execution cost. The identification of mammals through this method is possible using the microstructural patterns of the cuticle scales and the analysis of hair medullary cells. These methods also enable to classify order, family, gender, and species [5]. 

The morphometric comparison of microstructures in the surface relief of hair is another method proposed for hair microstructural patterns analysis, with focus on cuticular and medullary anatomy [6]. This study was carried out aiming to identify the bovine breeds Caracu, Curraleiro Pé-Duro, Pantaneiro (*Bos taurus taurus*), and Nelore (*Bos taurus indicus*) through trichological morphology and morphometry, and to validate this technique by comparing the findings with molecular genetic characterization results of the same animals. 

## 2. Materials and Methods 

Morphological and morphometric characteristics of guard hairs were evaluated from 40 animals of the breeds Caracu, Curraleiro Pé-Duro, Bovino Pantaneiro, and Nelore. A total of 160 animals of both sexes and from different geographic regions and herds: Curraleiro Pé-Duro ● = 16° 47’ 15” S, 49° 00′ 0” W; Nelore and Caracu ■ = 20° 27’ 07” S, 54° 42’ 59” W; and Bovino Pantaneiro ▲ = 16° 47’ 4.9” S, 56 56’ 56” W; 18° 59’ 11” S, 56° 37’ 19” W; 19° 57’ 6.8” S, 54° 42’ 19.8” W were selected to ensure a representative sample of racial groups. Because it is a component project of a research network, it used samples collected from herds whose owners, public or private, are conscious collaborators of these activities. Therefore, it is affirmed that they agreed to all the procedures performed.

With latex gloves, hair samples were manually collected from the animals on the region between the shoulders blades, that is, on the intersection of the longitudinal median line of the body with the scapula line [7]. The hair was pulled out with the fingertips, without required another instrument. No manifestation of pain or discomfort was observed, the method of collection is fast and non-invasive. The individual mechanical containment was done in a chute for activity restriction, without the need to squeeze the animal. The procedures performed by researchers of the Pro Centro Oeste Research Network followed the criteria of well-being and ethics in the use of animals, described by World Organization for Animal Health—OIE.

Hundreds of hairs were stored in small paper envelopes, labeled with data on the species, collection site, date of collection, and sex of the individuals [7]. Hundreds of hairs were stored in small paper envelopes, labeled with data on the species, collection site, date of collection and sex of the individuals [7]. These sampling has been made since 2011 and deposited in an official biological collection registered in the Genetic Property Management Council (Process 02000.002446/2011-65), to be used in genetic projects executed by the research network “Characterization, Conservation and Use of Brazilian Local Bovine Breeds: Curraleiro and Bovino Pantaneiro”. The material remained stored at room temperature at the Embrapa Pantanal Trichology Laboratory. 

The National Animal Experimentation Control Council—CONCEA authorized this research by the local Ethics Committee evaluation, registered as (CEUA/CPAP 0012/2016). 

### 2.1. Slides Preparation of The Cuticular Print and Medulla

Subsamples were separated from each individual until good-quality slides were obtained of five guard hairs. Only hairs that contained bulb, shaft, and shield were considered for the preparation of the slides. Slides were prepared at the Embrapa Pantanal Trichology Laboratory and at the Anatomy and Physiology laboratory of the Department of Comparative Biomedicine and Food (BCA) in the University of Padova, Italy. The procedures used in the preparation of slides for analysis of medulla and cuticle imprints were adapted from the methodology proposed in the literature [7,8].

### 2.2. Hair Morphological and Morphometric Analysis

Sections of the hair images were acquired digitally through an optical microscopy without distortion with 10.0× ocular, and 20.0 and 40.0× objectives with light transmitted without filtering (Nikon microscope model: Eclipse E200, serial number: 110560 at Embrapa Pantanal and Leica microscope, and model DMR DFC480^®^ at the Department of Biology of the University of Padova, Padova, Italy). High-quality digital images of the hair sections, resolution 2048 × 1536 pixels, were obtained with a digital camera and two capture programs, Motic Images Plus^®^ 2.0 ML and Leica IM500 Image Manager^®^. The photographic images were analyzed in a microscope with 200 and 400× magnification to coincide with a two-dimensional analysis in order to cover all hair thickness in the visual field.

The cuticular pattern in the shaft consisted of six scales characters (margin imbrication, shape, dimension, orientation, margin ornamentation, and margin continuity), with the total of 15 patterns. The medullary pattern in the shield consisted of six characters (presence, continuity, number of rows of cells, arrangement of cells, margin ornamentation and shape of medullary cells, with the total of 17 patterns, as described in the literature [9]. 

The cell structures inside the medulla, called vacuoles, are important for breed differentiation. The presence of these cells was counted in a range of 300 μm along the hair and the samples were classified in function of the number of vacuoles found: More than 11 vacuoles/300 μm “marked presence of vacuoles”; less than 11 vacuoles/300 μm “moderate presence of vacuoles”; and when they were not present “absent”. Later, this information was transformed into scores (0–2) to be evaluated together with the morphometric data aiming to discriminate the pattern of the different breeds.

The variation of the morphometric characteristics of the hair samples among the breeds was evaluated comparatively in order to reduce the subjectivity of the conventional process of morphological analysis of hair microstructures for each evaluated area (shaft and shield). For each hair sample, two photographs were selected, one of the shield and one of the shaft. The images were recorded in JPG format. The free software “ImageJ—Open software” (National Institutes of Health, Bethesda, Maryland, USA, (http://rsbweb.nih.gov/ij/)) was used for image analysis. This program enables the conversion of pixels to units of measure using a standard slide. The photographs were converted to shades of gray to eliminate the color effect, particularly in the cuticle imprints, since it is relevant that the outline of the scales is well defined to make possible the image processing [6]. 

The methodology described by Tormen [6], with some adaptations for the measurements of hair cuticle patterns, was applied using the ImageJ software. The method consists in dividing the hair shaft into six sections of 50 μm along the hair length in order to obtain the quantitative characteristics of the scales from the hair cuticle with a completely or partially differentiation. Perimeter and area in μm and μm^2^, respectively, were measured for each structure recognized. The distortions due to a 3D analysis of the structure as a two-dimensional surface were considered uniform for all samples.

On the assumption that there is metric relation between the area and perimeter of a geometric (though not regular) shape, it is possible to use a new area and perimeter derivative value in the form of an index (EC). In this current study, the formula EC = area/perimeter^0.5^ was used. This transformation was performed to obtain a normalization of the data distribution (data correction factor). The methodology described by Felix et al. [7] was applied for assessment of the medullary patterns in the shield. After calibration of the software, measurements were taken to obtain the area (μm^2^) values. Medulla and medullary cortex characteristics of each hair were measured six times: Medullary diameter (MD), medullary cortex (MC), and hair diameter (HD). For both cuticle and medulla assessments, hair samples were placed on slides with the distal part facing the operator’s left and the screenings always occurred from left to right.

### 2.3. Genetic Characterization

The genetic characterization was performed in the Laboratory of Applied Molecular Genetics of the Animal Breeding Consulting Company S.L. (ABC S.L) at the University of Cordoba in Spain. Using DNA extracted from hair samples, Caracu (*n* = 37), Nelore (*n* = 40), Curraleiro Pé-Duro (*n* = 39) and Bovino Pantaneiro (*n* = 39) were genotyped with sixteen microsatellite markers: *BM1314, BM1818, BM1824, BM2113, CRSM60, ETH003, ETH010, ETH185, ETH225, ILSTS006, INRA023, INRA063, SPS115, TGLA122, TGLA126 and TGLA227*. They were amplified by polymerase chain reaction (PCR) in multiplex reactions and the fragments were separated in an ABI377XL automated sequencer (Applied Biosystems, Foster, CA, USA). 

The allelic classifications were performed using the computer programs, GENESCAN ANALYSIS 3.1.2 and GENOTYPER^®^ 2.5.2, respectively. Allele frequencies were estimated by direct counting. The tests were done based on an admixture model in which the allelic frequencies were correlated applying burn-in period of 50,000 and 500,000 iterations for data collection. One to four inferred clusters were performed with ten independent runs each. The best *K* value was obtained with the Evanno method [10] using the STRUCTURE HARVESTER 0.6.94 program that provided a graphic display.

### 2.4. Statistical Analysis

A completely randomized design was applied in the current study and an analysis of variance was used to test the fixed effect of breed on the morphometric measurements of the hair microstructures. The statistical differences were compared by the Tukey test (*p* < 0.05). Nine variables were used for the multivariate analysis; six of them were extracted from the morphometric data (area, perimeter, EC, DP, DM and CM) and the remainder from the morphological data (ORT_CUT = cuticle orientation, LG_MD = medulla width, and VAC = Vacuole). 

These variables were used to describe the microstructures of the guard hairs and thus, to perform the trichological characterization of each sample. Only the morphological characters that presented differences in the specific patterns were used in the multivariate analysis. It is worth mentioning that the combined analyses of morphometric and morphological information were reported as “trichological analysis”.

The statistical package vegan was used to observe the relationship between the different microstructural characteristics of the guard hairs using a Principal Component Analysis (PCA). Linear discriminant analysis was used to verify the ability of the trichological methodology to discriminate the bovine breeds. Also, the proportion of individuals classified within each group obtained with the trichological analysis was compared to the proportion of animals identified by using genetic analysis of population. The association between the matrix of the trichological discriminant analysis and the matrix of the genetic structure analysis was estimated using the Pearson correlation coefficient (*r*), adopting the Euclidean distance with standardized data, as a measure of dissimilarity. All analyses were performed using the R version 3.2.4 [11].

## 3. Results 

### 3.1. Hair Morphological Analysis

Through the combination of six characters described by Quadros and Monteiro-Filho [9], the breeds cuticular scales patterns were described, as detailed in Table 1. 

In general, a paved imbrication of the cuticle scales margins was the pattern observed for all breeds. This pattern is composed of cuticular scales that do not have free edges and there is no overlap between the edges of the adjacent scales. According to Quadros and Monteiro-Filho [9], this type of pattern resembles tiles on the floor or tiles on the wall. A wavy pattern was found when analyzing the shape of the scales for all breeds. In this type of pattern, the scale has no defined angle, its contour is waveform and composes a set of smooth transitions between protrusions and recess of varying depths [9]. 

**Figure 1:**
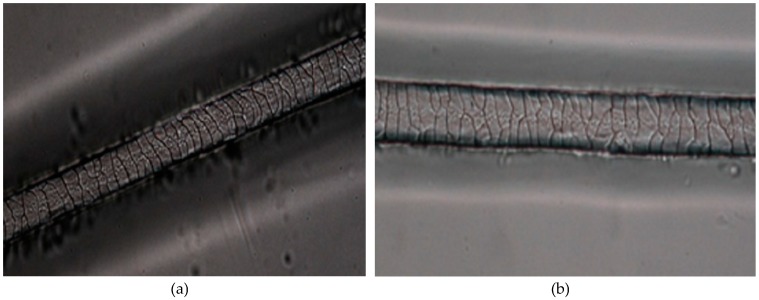
(**a**) A transverse wave cuticle pattern with smooth scales margins found on the hair shaft of the Brazilian local cattle Curraleiro Pé-Duro and Bovino Pantaneiro; (**b**) A irregular wave cuticle pattern with smooth scales margins found on the hair shaft of the Brazilian local cattle Caracu and zebu Nelore. Sections of the hair images were acquired digitally through an optical microscopy with 40.0× objectives.

When evaluating the cuticle characteristics related to “scales orientation”, it was possible to distinguish two animal groups, the first one was formed by the Curraleiro Pé-Duro and Bovino Pantaneiro breeds and the second one by Caracu and Nelore. A transversal pattern was obtained within the Curraleiro and Pantaneiro group (Figure 1a). For Nelore and Caracu, the pattern was identified as irregular (Figure 1b), similar to that described for *Bos taurus* and *Bos gaurus* [12].

**Figure 2:**
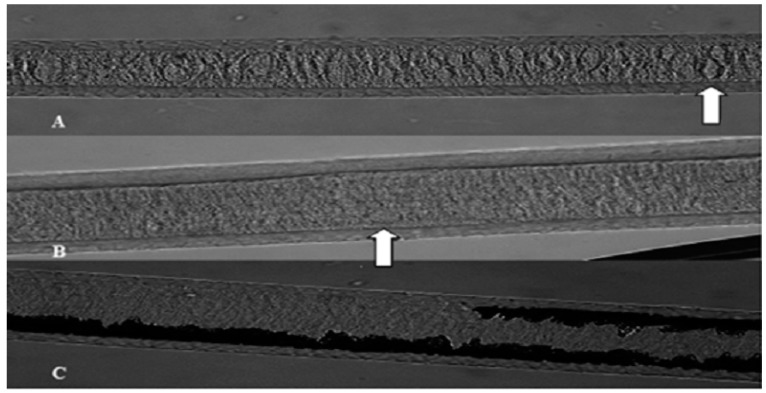
(**A**) Medulla pattern in the hair shield of Bovino Pantaneiro breed (continuous, multiseriate, anastomosed, trabecular with fimbriated margins and a marked presence of vacuoles); (**B**) medulla pattern in the hair shield of Caracu and Curraleiro Pé-Duro breeds (continuous, multiseriate, anastomosed, trabecular with fimbriated margins and a moderate presence of vacuoles); (**C**) medulla pattern in Nelore zebu breed (continuous, multiseriate, anastomosed, trabecular with fimbriated margins and no vacuoles).

Overall, the pattern for Caracu, Curraleiro Pé-Duro, Bovino Pantaneiro and Nelore breeds was described as continuous, multiseriate, anastomosed, and atrabecular with fimbriated margin. The “presence of vacuoles” was a determining characteristic for bovine breed differentiation (Figure 2). 

The counting of these vacuoles enabled a better description of the medullary form of the breeds and improved the discriminatory power of the method. The Caracu and Curraleiro Pé-Duro breeds had a moderate presence of vacuoles, Bovino Pantaneiro had a marked presence and Nelore was classified as absent. One of the most relevant subjects is that the medullary characteristics were important for identification of bovine breeds in this study. Thus, it was possible to differentiate all bovine breeds by the association of medullary patterns with cuticular patterns.

### 3.2. Hair Morphometric Analysis

Cuticle characteristics comparisons are presented in Table 2. When evaluating the data related to the measurements of bovine hairs, the model using breeds was significant for all response variables (*p* < 0.001).

Using the three morphometric variables only—hair diameter (HD), medullary diameter (MD) and medullary cortex (MC)—it was not possible to separate the breeds into specific groups. In particular, the results found for medullary cortex (CM) corroborated the morphological diagnoses of the medullary patterns because Caracu and Curraleiro Pé-Duro presented the same pattern: Continuous, multiseriate, anastomosed, trabecular with fimbriated margin and a moderate presence of vacuoles. It is worth mentioning that, the size of the cortex in relation to the medulla has been used successfully in the identification of species [13].

In the measurement of the area and perimeter of cuticle scales, the breed effect was significant (*p* < 0.001), as indicated in Table 3. Although the EC variable was used to obtain normalization in the distribution of the data and its use reduced the coefficient of variation of the measures related to the shape of the cuticle scales, it did not provide greater discrimination than that obtained using the variables separately.

### 3.3. Trichological Analysis

The results of the discriminant analysis showed that the unioncuticle and medulla patterns of hair (Table 4) was more accurate in differentiating the breeds than when the information was evaluated separately (Table 3) or when only the form of these microstructures was considered (Table 1). The results corroborated the findings of literature [9] who stated that it is possible to identify a species through the combination of the cuticular, medullary and cortical patterns of the mammalian guard hairs, since this combination gives diagnostic characteristics to a specific species. Therefore, it is possible to say that for breeds identification, the combination of patterns and measurements of these microstructures could be associated.

After studying the information obtained by analyzing the morphological and morphometric data of guard hairs microstructures separately, the data were analyzed through multivariate methods. This decision was made based on the fact that the information obtained by univariate analyses can often be incomplete, especially when there is a correlation between the variables.

The use of multivariate analysis allows combining multiple information from the experimental unit [14]. When the number of characteristics is elevated, many of them may contribute little to the discrimination of the individuals and this situation increases the characterization task, but without improving precision, making the analysis and interpretation of the data more complex [14]. Moreover, with the use of multivariate analysis, one can eliminate redundant and difficult-to-measure characteristics, reducing the time and costs of experiments.

**Figure 3:**
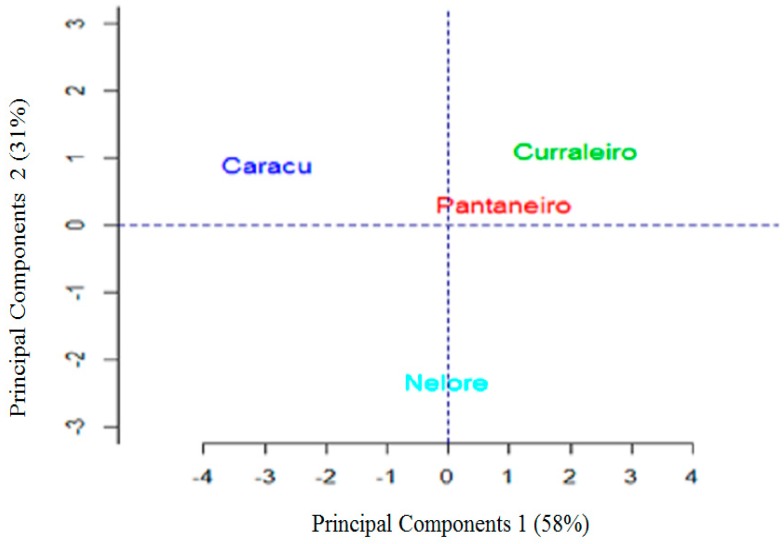
Graphic dispersion of the first two principal components to observe the differences between the breeds (Caracu, Curraleiro Pé-Duro, Bovino Pantaneiro, and Nelore).

The analysis of principal components showed that the first two components together explained 89% of the variation of the data derived from the nine characteristics studied (Figure 3). 

When analyzing the graphical dispersion shown in Figure 3, it is evident that Curraleiros Pé-Duro and Bovino Pantaneiro cattle are more similar. PCA results confirmed what has previously been described on the local Brazilian breed, that is, Caracu has the greatest differentiation. The genetic improvement work, for both meat and milk, to which Caracu was submitted, is the most probable justification for the divergence observed. The zebu breed showed greater trichological differentiation, since among all the breeds these are the ones that are more distant in the graph. The results corroborated the findings of literature [15] that found genetic divergence between locally adapted breeds (*Bos taurus taurus*) and zebu breeds (*Bos taurus indicus*) using SNP markers.

### 3.4. Genetic Characterization

**Figure 4:**
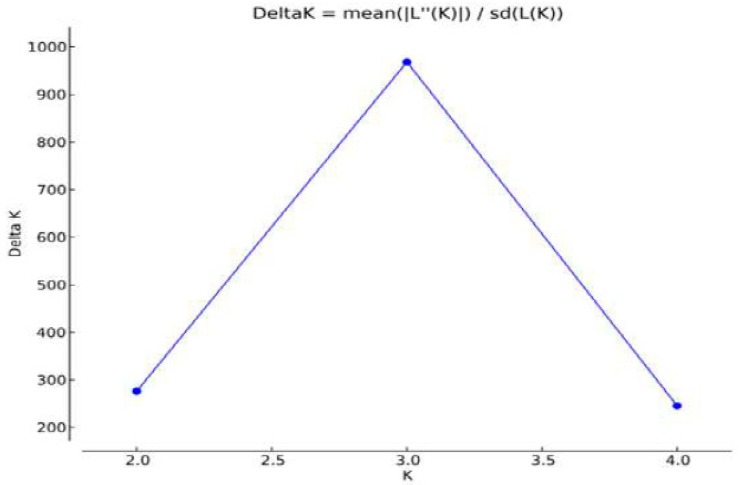
Magnitude of K as a function of K given by the average of 5 independent runs with 500,000 interactions each and x burn-in period. The value that stands out represents the real value of K, that is, the number of inferred populations.

**Figure 5:**
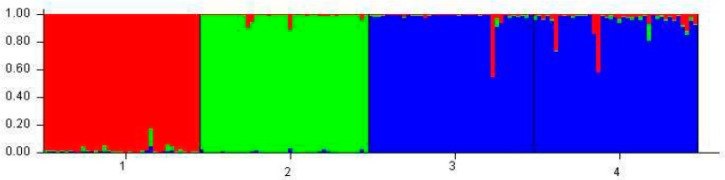
Individual grouping of 40 individuals from the four bovine breeds inferred by the Bayesian statistical method using the STRUCTURE program. Each of the 160 animals is represented by a vertical line divided into segments ranked according to color and size corresponding to the relative proportion of the genome of the animal corresponding to a particular cluster. Different breeds are separated by black lines. 1 = Caracu; 2 = Nelore; 3 = Curraleiro and 4 = Bovino Pantaneiro.

For the genetic characterization based on the graph of Figure 4, it is possible to observe that the real number of k given as a function of the magnitude of ΔK was three. The Caracu breed, although also a local breed, is already distinguished for its history of selection and breeding from the others [16], as well as the zebu breed (Figure 5).

In spite of this, the common origin of the Caracu breed with the others locally adapted breeds can be observed by the presence of common alleles in some animals of the Curraleiro Pé-Duro/Bovino Pantaneiro group. This result corroborates that found by the principal components analysis described in Figure 3, pointing out that the trichological analysis is an efficient method from the point of view of the classification of individuals within breed groups. 

For this study it was assumed that 16 microsatellite markers would be enough to differentiate four bovine breeds. However, as the local Brazilian breeds Curraleiro Pé-Duro and Bovino Pantaneiro were very close to each other, this distinction was not possible. Probably these breeds would have been differentiated if a larger number of markers had been used. However, as the objective of the genetic characterization through DNA markers in the present study was to validate the methodology of trichological analysis for breed distinction, Table 5 presents the probabilities of identifying individuals from the four genetic groups sampled. 

It was also observed in both trichological and genetic analyses (Table 3 and Table 4, respectively) that in the Curraleiro Pé-Duro breed, some individuals also presented allelic sharing as a function of the common origin, which was more apparent with the Caracu breed. For Caracu and Nelore breeds, the genetic results matched those presented by the trichological analysis as well.

The trichological method for differentiation of bovine breeds is robust, since the analysis recognized differences in individuals of groups as captured by the genetic analysis. The same animals, which were described as being from another group by the genetic method, were also characterized by trichology, demonstrating the richness of the proposed method for breeds identification.

Precision of the trichological analysis were further confirmed by the comparison of the population genetic structure analysis with the results of the discriminant analysis through Pearson’s correlation test. However, for this comparison, it was necessary to remove from the trichological analysis the animals that were not genetically characterized in order to avoid erroneous results.

Thus, in Table 6, the trichological data concerning the proportions of individuals classified correctly within each breed were described as Caracu (*n* = 37), Nelore (*n* = 40), Curraleiro (*n* = 39) and Pantaneiro (*n* = 39). These were used to generate the matrix that served as a comparative parameter with the genetic data, in order to verify if the data were associated.

Pearson correlation test showed that there was a high positive and significant correlation between matrices (*r* 0.996 and *p* < 0.001). The results corroborate the findings of literature described before by other authors [17] in studies for compared methods of morphological and molecular identification of Neotropical felid species through fecal samples, and the efficiency of these methods in identifying species.

## 4. Discussion

### 4.1. Hair Morphological Analysis

Quadros and Monteiro-Filho [9] stated that the cuticular scales with a transverse pattern are oriented transversely to the longitudinal axis of the hair. This type of cuticular pattern (transverse wavy pattern) cannot be used for species differentiation, since it is common to several orders of mammals [16]. In the irregular pattern, the scales can be either transverse or oblique, and even longitudinally, towards the largest axis of the hair. In addition, the size of the scales may also vary [9]. Although Keogh [17] and Teerink [18] described in their studies four types of ornamentation for species differentiation and, also, Quadros and Monteiro-Filho [6] reported that the characteristic ornamentations may be interesting for this purpose, in the present work no ornamentation was observed and all breeds showed scales with smooth margins.

De Marinis and Asprea [19] reported that the domestication process generated changes in several of the cuticular characteristics and its resulting homogeneity compromises the identification of the species. Some similarity between the characteristics studied for these local breeds was expected and such similarities were found between Curraleiro Pé-Duro and Bovino Pantaneiro. Nevertheless, similarities were unexpected between *Bos taurus taurus* (Caracu) and *Bos taurus indicus* (Nelore). One cannot affirm, but it is probable that the changes could have occurred with the cattle of this breed caused by selection programs and genetic improvement imposed on Caracu breeds to enhance characteristics related to meat and milk yields [19].

In comparison with other studies, De Marinis and Asprea [19], without citation of breeds, reported some characteristics similar to the patterns observed in this study. The authors stated that the scales are presented transversely with distant margin and irregular wave scale. Conversely Sari and Arpacik [20] found irregular rippled scale patterns can be observed in all studied species except the Bovidae family and wild boar, and cited that this structure in the entire hair length of the Bovidae family and wild boar is regularly waved.

However, there are divergences in the nomenclature used by De Marinis and Asprea [19] and Quadros and Monteiro-Filho [9] in which this study is based on. For Quadros and Monteiro-Filho [9], the scales orientation of guard hairs can be transverse or irregular, that is, the cuticle scales did not present the two patterns as suggested by De Marinis and Asprea [19], unless this occurs in different portions of the hair (shaft and shield, for example).

Breed differentiation in two groups by scales assessment was only possible because Curraleiro Pé-Duro and Bovino Pantaneiro breeds presented a transverse pattern, whereas the group Caracu and Nelore an irregular pattern. It was not possible to conclude that this pattern is a more common characteristic for European bovine breeds since the study by De Marinis and Asprea [19] did not specify the breed or breeds used to represent the bovine species. In fact, this was not the objective of the aforementioned authors but to distinguish wild ungulates from domestic ones by hair characteristics.

The cuticle morphological pattern studied here did not match the characteristics described in the literature [21]. These authors described that the cuticle scales of *Bos taurus taurus* are flat and deeply imbricated, arranged at an angle oblique to the hair longitudinal axis and with margins presenting a crenated appearance. Deedrick and Koch [22] also described bovine cuticle scales as being imbricated and without protrusions on the hair shaft.

The medullary pattern observed in the guard hairs of Brazilian bovine breeds has been described before by other authors in studies for nomenclature proposals [9,18,23,24], in biological studies about eating habits of carnivores through fecal analysis [16], in the identification of prey mammals and predators [25], in studies of Brazilian felines [26], in studies of species with forensic interest, in food quality control [5], and for ecological studies [27].

For Chernova [27], the different patterns of medullary cells may be expressions of the evolution of the species. In addition, Perrin and Campbell [28] and De Marinis and Asprea [19] reported that biotic and abiotic ecological factors also played an important role in determining morphological patterns of hair.

Overall, the pattern for Caracu, Curraleiro Pé-Duro, Bovino Pantaneiro, and Nelore breeds was described as continuous, multiseriate, anastomosed, and trabecular with fimbriated margin. Literature is divergent about the morphological patterns of guardian hairs of species from the Bovidae family. This is because most of the literature found is focused on forensic research and not on differentiating and characterizing the bovine breeds. Gaudette [29] reported that in the Bovidae family there may be individuals who do not present a medullary structure along the hair, whereas others present a continuous or discontinuous medullary pattern. Deedrick and Koch [22] also described bovines hair without medulla or when present it was continuous.

It is difficult to compare the results of this study with those available in the literature since they only aimed to identify populations without specifying which gender or which breeds were used in these studies. Likewise, the lack of details on the portion of the hair used for observation compromises possible comparisons. For the same reasons, comparisons about hair width are uncertain.

In fact, Deedrick and Koch [22] and Gaudette [29] cited that this structure within a Family is relatively narrow. Conversely, De Marinis and Asprea [19] described that the width of the cortex represents half or 1/3 of the width of the medulla. Given these points, it is possible that the medulla analyzed by these authors varied from intermediate to narrow, since this structure characterizes the innermost layer of the hair, and it is superimposed by the cuticle and the cortex.

In the current study, a narrow medulla was found in only 10% of the hair within the breeds evaluated. In 90% of the bovines analyzed, the predominant pattern was the width that corresponds to that described by literature [12] for *Bos taurus taurus*. De Marinis and Asprea [19] report that domestic ungulates may have uniseriate and multiseriate medullary structures, whereas bovines have the latter one. The uniseriate pattern was not observed in the current study, that is, along the shield of the guard hair was observed a continuous medulla with multisserie rows of cells in its width with two or more rows of cells present.

The disposition of the cells inside the medulla in bovine breeds was described by Quadros and Monteiro-Filho [6] as anastomosed because there were fusions between the cells forming cellular arrangements that can define spaces of the cortex with variations in shape and size. Regarding the way in which the cell can be present along the shield, all bovines were classified as trabecular, that is, the cells are flattened as septa or trabeculae and are arranged close and parallel to each other, but transversally to the largest axis, and with longitudinal anastomoses that ligate these parallel trabeculae [6].

In all hairs, the margin ornamentation of the medulla along the shield was identified and described by Quadros and Monteiro-Filho [6] as a fimbriae or fimbriated pattern, that is, the occurrence of many protrusions and narrow recesses, but with variable depths and irregular distribution along the margins, constituting what appears to be a fringe.

During the evaluations of the guard hairs (although all bovines had the same trabecular pattern), it was possible to visualize some circular or oval structures in three of the analyzed breeds, except for Nelore. These structures were called vacuoles and according to the quantity were defined in categories: Marked, moderate and absent. The vacuoles abundance was a determining characteristic for bovine breed differentiation. The same was previously described by De Marinis and Asprea [19]; however, in this study, these cell structures were counted when they were present inside the medulla. The counting of these vacuoles enabled a better description of the medullary form of the breeds and improved the discriminatory power of the method. Thus, Caracu and Curraleiro Pé-Duro breeds had a moderate presence of vacuoles, Bovino Pantaneiro had a marked presence and Nelore was classified as absent.

Quadros and Monteiro-Filho [25] reported that, in general, the cuticular characters are the most important for species differentiation, whereas medullary characters are relevant to describe Families and zoological Orders. It is noteworthy that these characteristics are also useful in studies related to the identification of domestic breeds as long as they are analyzed together.

Although the differentiation was possible, this type of analysis did not seem to be the most adequate to determine the microstructural patterns of both cuticles and bovine medulla due to its subjectivity. In addition, the structures forms were all very similar, besides the similarity with other mammalian species already described by other authors [5,9,12,18,24,25,26,30].

De Marinis e Agnelli [31] mentioned that in microscopic morphology the use of medulla structure has priority over the cuticle pattern and the use of the cuticle pattern individually will cause confusion, but Sari & Arpacik [20] reported that at lower levels, such as subfamily and genus, the medulla structure does not give the correct results in species identification due to its high resemblance, so the cuticle pattern (trichological analysis) was used for identification in all species.

### 4.2. Hair Morphometrical Analysis

The hair comparison analysis is essentially subjective, and despite taking into account the expert opinions, the results and conclusions are not quantifiable yet [32]. For this reason, in order to reduce the subjectivity in this study, not only the morphological characteristics but also the morphometric features of the hairs microstructures were determined.

Serrano et al. [33] and Egito et al. [34,35] mentioned that there was genetic introgression from zebu breeds in local cattle including Bovino Pantaneiro cattle. The authors stated that in the Bovino Pantaneiro breed this introgression of alleles of zebu origin was verified due to probable miscegenations that occurred in the past. Thus, there is a smaller divergence of Bovino Pantaneiro in relation to the Nelore breed.

In a previous study about the cytogenetic and molecular analysis of the Bovino Pantaneiro breed [36], the authors reported that among the Bovino Pantaneiro group analyzed, there was the presence of Y chromosome of zebu (acrocentric), which suggests racial contamination by zebu cattle, since the Pantaneiro breed comes from taurine (submetacentric) cattle. Among the locally adapted Brazilian breeds, Caracu showed the greatest differentiation in both morphological and morphometry features of the cuticle. These results corroborated those described in previous studies [15,33,34], when comparing with molecular markers of the population structure of locally adapted Brazilian bovine breeds.

### 4.3. Genetic Characterization

As in this study, in the genetic evaluation, the Caracu breed is easily distinguished from the others, because it possesses the lowest allelic richness, probably due to the genetic improvement by the breeders’ association [34] and probably due to its formation and objective of selection (milk yield) since 1893 [15,37]. The results of this study can also be justified considering the history of formation of these populations from their introduction in Brazil by the colonizers, since the probable ancestor of the Curraleiro Pé-Duro and Bovino Pantaneiro breeds was *Bos taurus ibericus*, whereas Caracu had as an ancestor *Bos taurus aquitanicus*. Ginja et al. [38] reported that historical information indicates that Creole breeds have their own identity and a fingerprint unique to this group. The genetic legacy of Iberian cattle is still represented in Creoles, but other influences can also be detected. The Nelore’s easy identification by different methods is completely justifiable and expected because they have Indian origin (*Bos taurus indicus*).

The results for area and EC are consistent with those obtained in literature [33] based on patterns of diversity and similarity obtained for the same bovine breeds, however using DNA markers (RAPD) and also with the study using microsatellites markers [34]. Both studies mentioned that there was genetic introgression from zebu breeds in local cattle including Bovino Pantaneiro cattle. Considering population structures, it was possible to observe that the Curraleiro and Bovino Pantaneiro have a large number of shared alleles that place them in the same population.

After studying the information obtained by analyzing the morphological and morphometric data of guard hairs microstructures separately, the data were analyzed through multivariate methods. This decision was made based on the fact that the information obtained by univariate analyses can often be incomplete, especially when there is a correlation between the variables.

The results observed indicated that genetic and trichological techniques are similar in terms of individual classification within breed groups and because of that, the trichological analysis can be considered useful as a bovine cattle breed marker. Alberts et al. [39] reported that, this allows more freedom in the choice of approaches, depending on the objective of the study. If one seeks more flexibility and lacks time and/or more elaborate laboratory resources, or needs a result when still in the field, one should choose trichology. If, on the other hand, one requires absolute accuracy and/or has the time and access to a genetics laboratory, the molecular method is the appropriate option.

## 5. Conclusions

We conclude trichological analysis is a useful method for cattle breed identification. Its potential use for other mammal species of interest for animal production should be studied because is a simple, low-cost, and non-invasive method that can be even used as a breed traceability tool.

## Figures and Tables

**Table 1:** Synopsis of morphological patterns used in the identification and differentiation of the bovine breeds studied.

Cuticle and Medullar Patterns	Breeds			
	Caracu	Curraleiro	B. Pantaneiro	Nelore
Margins imbrication	Paved	Paved	Paved	Paved
Scales shape	Wavy	Wavy	Wavy	Wavy
Scales orientation	Irregular	Transverse	Transverse	Irregular
Ornamentation of scales margins	Smooth	Smooth	Smooth	Smooth
Medulla presence	Present	Present	Present	Present
Medulla continuity	Continuous	Continuous	Continuous	Continuous
Cells rows	Multiseriate	Multiseriate	Multiseriate	Multiseriate
Cells disposition	Anastomosed	Anastomosed	Anastomosed	Anastomosed
Cells shape	Trabecular	Trabecular	Trabecular	Trabecular
Ornamentation of medulla margins	Fimbriate	Fimbriate	Fimbriate	Fimbriate
Vacuoles presence	Moderate	Moderate	Marked	Absent

**Table 2:** Analysis of variance and comparison test of the means of the parameters of cattle hairs of Bovino Pantaneiro, Curraleiro Pé-Duro, Caracu, and Nelore.

Cuticle and MedullaVariables				
Variables	Breeds *	Mean (µm)	Standard Deviation	CV (%)
Area	Caracu	305.75 ^a^	53.63	17.54
	Bovino Pantaneiro	254.31 ^b^	48.83	19.20
	Nelore	233.82 ^b^	38.47	16.45
	Curraleiro Pé-Duro	199.00 ^c^	30.35	15.25
Perimeter	Caracu	87.96 ^a^	9.53	10.83
	Bovino Pantaneiro	80.68 ^b^	10.57	13.10
	Nelore	74.47 ^c^	7.55	10.14
	Curraleiro Pé-Duro	69.09 ^d^	6.39	9.25
EC	Caracu	1.86 ^a^	0.07	3.80
	Bovino Pantaneiro	1.77 ^b^	0.07	3.68
	Nelore	1.77 ^b^	0.06	3.44
	Curraleiro Pé-Duro	1.69 ^c^	0.05	3.15
HD	Pantaneiro	101.42 ^b^	18.67	18.41
	Curraleiro Pé-Duro	108.43 ^b^	29.55	27.25
	Caracu	121.25 ^a^	22.95	18.93
	Nelore	107.19 ^b^	26.32	24.55
MD	Bovino Pantaneiro	61.49 ^b^	15.53	25.26
	Curraleiro Pé-Duro	62.39 ^b^	18.26	29.27
	Caracu	75.19 ^a^	19.63	26.11
	Nelore	78.70 ^a^	24.91	31.65
MC	Bovino Pantaneiro	20.14 ^b^	4.50	22.34
	Curraleiro Pé-Duro	22.98 ^a^	6.29	27.37
	Caracu	22.93 ^a^	6.40	27.92
	Nelore	15.42 ^c^	4.26	27.62

* EC = cuticular scale index; CV = Coefficient of variation; HD = hair diameter; MD = medullary diameter; MC = medullary cortex; Means with different letters among breeds are statistically significant by the Tukey test (*p* < 0.05).

**Table 3:** Discriminant analysis with number of observations and percentage of guard hair classification of cattle Bovino Pantaneiro, Curraleiro Pé-Duro, Caracu and Nelore.

Breeds		Caracu	Nelore	Curraleiro	B. Pantaneiro	Total
Number of observations and percentage of classification by cuticle						
**Caracu**	n°	24	8	2	6	40
	%	(60.00)	(20.00)	(5.00)	(15.00)	100
**Nelore**	n°	4	21	8	7	40
	%	(10.00)	(52.50)	(20.00)	(17.50)	100
**Curraleiro**	n°	0	8	28	4	40
	%	(0.00)	(20.00)	(70.00)	(10.00)	100
**Pantaneiro**	n°	8	9	8	15	40
	%	(20.00)	(22.50)	(20.00)	(37.50)	100
**Total**	n°	36	46	46	32	160
	%	(22.50)	(28.75)	(28.75)	(20.00)	100
Global mean error		0.400	0.475	0.300	0.625	0.450
**Number of observations and percentage of classification by medulla**						
**Breeds**		**Caracu**	**Nelore**	**Curraleiro**	**B. Pantaneiro**	**Total**
**Caracu**	n°	17	6	11	6	40
	%	(42.50)	(15.00)	(27.50)	(15.00)	100
**Nelore**	n°	0	29	3	8	40
	%	(0.00)	(72.50)	(7.50)	(20.00)	100
**Curraleiro**	n°	9	1	17	13	40
	%	(22.50)	(2.50)	(42.50)	(32.50)	100
**Pantaneiro**	n°	9	7	8	16	40
	%	(22.50)	(17.50)	(20.00)	(40.00)	100
**Total**	n°	35	43	39	43	160
	%	(2188)	(26.88)	(24.38)	26.88	100
**Global mean error**		0.575	0.275	0.575	0.600	0.506

**Table 4:** Discriminant analysis and percentage of classification obtained from the microstructural characteristics of the guard hairs (trichological analysis) of cattle Bovino Pantaneiro, Curraleiro Pé-Duro, Caracu and Nelore.

Number of Observations and Percentage of Classification Obtained of Trichological Analysis							
Breeds		Caracu	Nelore	Curraleiro	Pantaneiro	Total
**Caracu**	n°	38	2	0	0	40
	%	(95.00)	(5.00)	(0.00)	(0.00)	100
**Nelore**	n°	4	36	0	0	40
	%	(10.00)	(90.00)	(0.00)	(0.00)	100
**Curraleiro**	n°	0	0	37	3	40
	%	(0.00)	(0.00)	(92.50)	(7.50)	100
**Pantaneiro**	n°	0	0	3	37	40
	%	(0.00)	(0.00)	(7.69)	(92.31)	100
**Total**	n°	42	38	40	40	160
	%	(26.42)	(23.90)	(25.16)	(24.52)	100
**Global mean error**		0.050	0.100	0.075	0.076	0.075

**Table 5:** Proportion of individuals from each of the four genetic groups of bovines analyzed in relation to the four populations inferred by the STRUCTURE program.

Genetic Groups	Breeds			
	Caracu	Nelore	Curraleiro	Bovino Pantaneiro
**Caracu**	0.972	0.013	0.006	0.009
**Nelore**	0.011	0.977	0.004	0.008
**Curraleiro**	0.022	0.007	0.892	0.079
**Bovino Pantaneiro**	0.015	0.007	0.024	0.954

**Table 6:** Discriminant analysis, number of observations, and percentage of classification obtained from the microstructural characteristics of guard hairs of 155 bovine breeds Caracu, Curraleiro Pé-Duro, Pantaneiro and Nelore (trichological analysis).

Breeds		Caracu	Nelore	Curraleiro	B. Pantaneiro	Total
**Caracu**	n°	35	2	0	0.	37
		0.946	0.054	0.000	0.000	100%
**Nelore**	n°	4	36	0	0	40
		0.100	0.900	0.000	0.000	100%
**Curraleiro**	n°	0	0	37	2	39
		0.000	0.000	0.948	0.051	100%
**B. Pantaneiro**	n°	0	0	2	37	39
		0.000	0.000	0.051	0.948	100%
**Total**	n°	39	38	39	39	155
		0.252	0.245	0.252	0.252	100%
**Global mean error**		0.050	0.100	0.075	0.076	0.075
